# Fast and customizable image formation model for optical coherence tomography

**DOI:** 10.1364/BOE.534263

**Published:** 2024-11-13

**Authors:** Andrea Mazzolani, Callum Macdonald, Peter R. T. Munro

**Affiliations:** Department of Medical Physics and Biomedical Engineering, University College London, Malet Place, Gower Street, London WC1E 6BT, UK

## Abstract

Optical coherence tomography (OCT) is a technique that performs high-resolution, three-dimensional, imaging of semi-transparent scattering biological tissues. Models of OCT image formation are needed for applications such as aiding image interpretation and validating OCT signal processing techniques. Existing image formation models generally trade off between model realism and computation time. In particular, the most realistic models tend to be highly computationally demanding, which becomes a limiting factor when simulating C-scan generation. Here we present an OCT image formation model based on the first-order Born approximation that is significantly faster than existing models, whilst maintaining a high degree of realism. This model is made more powerful because it is amenable to simulation of phase sensitive OCT, thus making it applicable to scenarios where sample displacement is of interest, such as optical coherence elastography (OCE) or Doppler OCT. The low computational cost of the model also makes it suitable for creating large OCT data sets needed for training deep learning OCT signal processing models. We present details of our novel image formation model and demonstrate its accuracy and computational efficiency.

## 1. Introduction

Optical coherence tomography (OCT) is an imaging tool [1] used widely in ophthalmology [2] and, increasingly, in other medical imaging applications such as dermatology [3], cardiology [4] and gastroenterology [5]. OCT is based on a combination of low coherence interferometry and confocal microscopy, which enables the recording of high-resolution depth profiles of semi-transparent scattering biological tissues. Models for OCT image formation employ the physics underlying OCT to simulate images possessing salient features consistent with images acquired experimentally. Such features include realistic speckle properties, focal plane generation, resolution, depth of focus and sample induced aberration. Simulation of OCT image formation has proven powerful for investigating and developing a range of OCT-based imaging modalities, such as optical coherence elastography (OCE) [6,7] and optical coherence tomography angiography (OCTA) [8].

In the last two decades, several methods of simulating OCT image formation have been developed [8–21]. The usefulness of such image formation models is determined by how accurately a sample can be, and needs to be, represented and by the degree of approximation of the model of light propagation. Making a rigorous error estimation of such models against experimental OCT data is challenging. One reason for this is that sample parameters, such as the microscopic refractive index distribution, which are integral to OCT image formation are, in general, not known with sufficient accuracy to simulate the experiment.

Image formation models can be classified as either analytical or numerical. Analytical models are based on mathematical functions describing the response of the optical system, usually the point-spread function (PSF) [8–10]. The advantage of these models is that they are generally computationally inexpensive and they may provide additional information regarding the physical properties of the imaged object. However, because of the intrinsic complexity of light scattering, significant approximations are often necessary to find such analytical descriptions when considering anything other than trivial samples.

Numerical models have been developed for scenarios where the governing equations are not amenable to analytical solution. The most common numerical models for image formation in OCT are based on Monte Carlo methods (MC) [11–14], full wave models (FW) [15–17,22,23] and numerical PSF models [18–20]. MC methods usually employ a particle description of light to model light-tissue interaction, where photon trajectories are simulated, therefore including multiple scattering. Their main weaknesses are that tissue is represented by statistically averaged properties and the computational cost can be high. FW models employ an electromagnetic description of light in three-dimensional space, by numerically solving Maxwell’s equations to determine how light is scattered by samples represented as three-dimensional refractive index distributions. FW models thus inherently include phenomena such as multiple scattering. Unlike MC methods, FW models can be used to simulate deterministic scatterers and wave properties of the illumination, including polarisation and coherence, for example. However, the computational cost of FW models can limit their applicability, particularly if a large number of OCT A-scans must be simulated or if the sample is too large. The available image formation models thus present a spectrum which varies in terms of realism and computational cost. For example, While analytical PSF based models are not detailed enough to generate realistic OCT B-scans or C-scans, the applicability of MC and FW based models can be limited by their computational cost.

However, a good compromise between computational cost and realism can be achieved by models employing a rigorous representation of the optical system, but assuming the first-order Born approximation to calculate the scattered field. This approximation is suitable in many applications where the primary contribution of the back-scattered field comes from single-scattering, as is often the case in OCT. Such a model, which does not make the paraxial approximation, and employs a rigorous vectorial description of the illumination [18,24], is faster than FW and MC methods, yet is still not fast enough for the applications targeted in this paper, such as the generation of OCT C-scans containing thousands of A-scans and hundreds of thousands of scatterers in minutes to hours. A model able to produce such C-scan datasets rapidly was proposed by Matveyev *et al.*, where the illumination was defined by a scalar Gaussian beam [8,19]. The same group recently proposed a generalisation of that model [20], which allows a more general description of the scalar illumination and does not make the paraxial approximation for the propagation of the beam, which must be calculated in advance on a transverse plane. The model calculates the scattered field efficiently by using angular spectrum decomposition and exploiting the speed of the fast Fourier transform (FFT) algorithm, which makes it suitable for the rapid simulation of C-scans. However, this model does not take advantage of redundancy when calculating a C-scans for samples with similar distributions of scatterers. In particular, an independent re-calculation is required when the positions of scatterers are perturbed.

In this paper we present a novel model of OCT image formation which is realistic and computationally efficient. Our model applies to samples composed of a discrete set of point scatterers and calculates the scattered field by employing the first-order Born approximation. The illumination is rigorously defined by the Debye-Wolf integral (DWI) [24,25], which allows the simulation of general vectorial beams, including aberrations. The numerical calculation of the DWI is highly computationally demanding for modelling OCT image formation, because it must be computed at each scatterer location and for each wavenumber of the simulation. We have overcome this computational bottleneck by using the Taylor expansion and a new optimal interpolation technique called *multi-spectral regression* (MSR). The Taylor expansion is employed to accurately approximate the DWI at all scatterer locations without directly numerically calculating the field at those points. Because of MSR, the model computation time does not scale with the number of wavenumbers and is memory efficient because the field is stored only for few wavenumbers. Scatterer distributions and lateral scanning can be implemented arbitrarily, without any restriction. This model can rapidly simulate C-scans for a broad range of OCT system parameters. The model is thus useful for analysing a range of phenomena observed in experiments and for training machine learning models that process raw OCT data. Furthermore, the model is extremely fast for simulating OCT C-scans for axially displaced scatterers, because the C-scan of the displaced scatterers is calculated as a perturbation to the initial C-scan, rather than requiring a complete recalculation. This is useful when modelling image formation in modalities such as optical coherence elastography (OCE) [26].

One example application is the simulation of large datasets of realistic OCT raw data used to train deep learning strain retrieval models for OCE, or to test the existing models. Within this example, we focus solely on an optical model of image formation in OCT which can be coupled to any model of sample deformation. In particular, we assume that users of this image formation model will also use a model of deformation appropriate to their application. This approach follows many works dating to the earliest OCT image formation model introduced by Schmitt [9], and later models such as [10] and [27]. Performing a rigorous accuracy analysis against multiphysics models, which include a mechanical model of the sample is thus beyond the scope of this paper.

We first overview models for OCT image formation based on the first-order Born approximation, focusing on the case where the illumination is rigorously defined by the DWI. Later, we explain in detail the two key components of our model, the Taylor expansion and the MSR. We first introduce the Taylor expansion based approximation and demonstrate its validity by comparing it with direct calculation of the DWI, including for phase-sensitive OCT, such as is used in elastography. We then introduce the MSR technique, which is employed to efficiently estimate arbitrary monochromatic components of the electromagnetic field from the computation of only few of components, which is useful for efficiently simulating OCT signals with hundreds of thousands of scatterers and several wavenumbers. Finally, we analyse the model’s accuracy and computation time when employing both approximations. We also show some examples of the model’s application, including the development of large OCT datasets that can be used for the training of deep learning models.

## 2. OCT image formation model

**Fig. 1.**
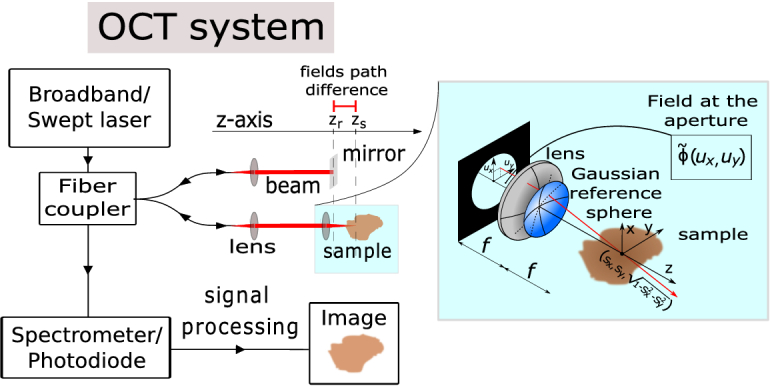
Diagram of a fiber-based OCT system as explained in section 2). The sample-arm fiber is depicted in the light-blue shaded section. The function $\tilde{\phi }\left(u_{x},u_{y}\right)$ specifies the profile of the field incident upon the aperture, which is mapped by the lens to the function $\phi \left(s_{x},s_{y}\right)$ on the Gaussian reference sphere. Each point $\left(u_{x},u_{y}\right)$ uniquely corresponds to the vector $\left(s_{x},s_{y},\sqrt{1-s_{x}^{2}-s_{y}^{2})}\right)$ , which defines the direction of a particular ray in the sample space (see [18] for more details).

A typical spectrometer based Fourier domain OCT system consists of a broadband source, an interferometer with reference and sample arms and a spectrometer (see Fig. (1)). A low coherence source emits light, which is split into the reference and sample arms. The reference arm beam is reflected by a mirror, while the sample scatters light back to the spectrometer, via the objective lens and optical fiber. For each wavenumber, k , the reference and sample-scattered fields are coupled into the respective fibers. This process is mathematically described by integration of the product of fields and fiber mode, over the end face of the fiber. We denote the results of such integrations as the mirror ( $\alpha_{mirr}(k))$ and sample ( $\alpha_{scat}(k)$ ) modal coefficients, respectively [16,28]. The detected current I(k) is then processed to create the OCT A-scan A(z) :

(1a) $$I(k):={\left\|\alpha_{scat}(k)+\alpha_{mirr}(k)\right\|}^{2}$$

(1b) $$A(z):=F^{-1}\left\{I(k)\right\}(2z)$$

where $F^{-1}\left\{.\right\}$ is the inverse Fourier transform operator. We assume that tissue can be represented as a collection of discrete scattering potentials, and the back-scattered field is calculated assuming the first-Born approximation. Each point scatterer is assumed to radiate as a harmonically oscillating dipole with moment proportional to the incident field, as shown by Born and Wolf [29]. Then, by denoting $E^{inc}\left(x_{s},y_{s},z_{s};k\right)$ as the monochromatic component of the incident electric field scattered by a scatterer at position $P_{s}=\left(x_{s},y_{s},z_{s}\right)$ with wavenumber k , the moment of the dipole which represents each scatterer, can be assumed proportional to the incident beam, i.e., $E^{scat}\left(x_{s},y_{s},z_{s};k\right)$ = $\rho_{s}E^{inc}\left(x_{s},y_{s},z_{s};k\right)$ , where $\rho_{s}$ is a positive coefficient representing the scattering cross-section of the scatterers, evaluated at $P_{s}$ . Signal attenuation can be simulated by varying $\rho_{s}$ as a function of the depth z . Since low numerical aperture objectives are employed in OCT, we assume that the longitudinal component of incident light can be neglected. Furthermore, without loss of generality, we assume that light incident upon the sample is linearly polarised in the x -direction. We point out, however, that the model can be easily adapted to cater for arbitrary polarisation states. Thus, assuming a sample composed by $N_{s}$ scatterers with positions $P_{s}=\left(x_{s},y_{s},z_{s}\right){|}_{s=1,\ldots N_{s}}$ , the modal coefficients of detected sample and reference arms can be described by [20,28]:

(2a) $$\alpha_{scat}\left(k\right):=\sum_{s=1}^{N_{s}}\rho_{s}{\left(E_{x}^{inc}\left(x_{s},y_{s},z_{s};k\right)\right)}^{2},$$

(2b) $$\alpha_{mirr}\left(k\right):=\Delta_{N}\sum_{t=1}^{N}\rho_{mirr}{\left(E_{x}^{inc}\left(x_{t},y_{t},z_{mirr};k\right)\right)}^{2},$$

where $E_{x}^{inc}$ is the x -component of the incident field. While Eq. (2a) represents the sum of the integrated fields back-scattered by each individual scatterer, Eq. (2b) represents the integral of the field reflected by the mirror, which is numerically calculated on the uniform grid of points $\{\left(x_{t},y_{t},z_{mirr}\right)\}{|}_{t=1,\ldots N}$ at the mirror plane $z=z_{mirr}$ with discrete step-size $\Delta_{N}$ , and $\rho_{mirr}$ is the reflectivity of the mirror (see Fig. (1)).

### 2.1 Scattered field definition

The most computationally costly aspect of OCT image formation models based on Eqs. (1–2) is generally the calculation of the incident field $E^{inc}\left(x_{s},y_{s},z_{s};k\right)$ . Matveyev *et al.* [20] modeled an arbitrary scalar incident field, assuming knowledge of the electric field at each point on the plane $z=z_{s}$ , before the region containing the scatterers, which is then used to calculate the field in all points of the computational space using angular spectrum propagation. In our approach, each monochromatic component of the electric field at point (x,y,z) , in the vicinity of the focus of the lens, is calculated using the DWI [24,25]. Assuming that the beam incident upon the aperture of the objective lens is linearly polarised in the x -direction, the focal field is given by:

(3) $$E(r,z;k)=-\frac{ikf}{2\pi }\iint_{\Omega }u\left(s\right)\frac{\phi (s;k)}{\sqrt{1-|s{|}^{2}}}e^{ik\eta \left(r\cdot s+z\sqrt{1-|s{|}^{2}}\right)}ds_{x}ds_{y}$$

where r=(x,y) , r⋅s is the dot product of the vectors r and s , f is the focal length of the objective lens, η is the refractive index in the focal region, $s=\left(s_{x},s_{y}\right)$ , $|s|=\sqrt{s_{x}^{2}+s_{y}^{2}}$ , ϕ(s;k) specifies the profile of the field on the Gaussian reference sphere of the lens, $\Omega =\left\{(s_{x},s_{y})\in R^{2}|\sqrt{s_{x}^{2}+s_{y}^{2}}<\frac{NA}{\eta }\right\}$ and u(s) is a vector which describes refraction by the lens of the field incident upon the Gaussian reference sphere of the lens, and is calculated using the generalized Jones matrix formalism [18]. For the remainder of this paper we will assume $\phi (s;k)=e^{-{\left(\frac{k}{W}\right)}^{2}\left(s_{x}^{2}+s_{y}^{2}\right)}$ , where W is a parameter that controls the waist radius of the Gaussian illumination (see Fig. (1)).

The DWI calculation can be sped up significantly by expressing it as linear combination of the Bessel functions of the first kind $J_{n}$ [30], which allows the field to be calculated partially analytically as [24,31]:

(4) $$\begin{array}{rl} & E^{inc}(r,z;k)=-ifk\int_{0}^{\arcsin\left(\frac{\text{NA}}{\eta }\right)}\sqrt{\cos(\theta )}\sin(\theta )e^{-{\left(\frac{k}{W}\right)}^{2}\sin^{2}(\theta )+i\eta kz\cos(\theta )}\\ & \;\cdot \left(\begin{array}{c} \left(1+\cos(\theta )\right)J_{0}\left(\eta k\rho \sin(\theta )\right)+\left(\cos(\theta )-1\right)\cos\left(2\psi \right)J_{2}\left(\eta k\rho \sin(\theta )\right)\\ \left(\cos(\theta )-1\right)\sin\left(2\psi \right)J_{2}\left(\eta k\rho \sin(\theta )\right)\\ 2\sin(\theta )\cos(\psi )J_{1}\left(\eta k\rho \sin(\theta )\right) \end{array}\right)d\theta \end{array}$$

where (ρ,ψ) are the polar coordinates of r . Even though Eq. (4) is an optimized version of the DWI, it is too slow to meet the objectives of this paper, where we aim to calculate thousands of A-scans. The high computational cost arises because Eq. (4) must be evaluated at each scatterer location and for each wavenumber of the sampled simulated spectrum.

## 3. Novel efficient model of OCT image formation

Our new OCT image formation model introduces a novel approximation to Eq. (4) which allows rapid evaluation of Eqs. (1–4). The new model enables very fast, phase-sensitive, OCT image formation simulations allowing several OCT C-scans to be evaluated, in the order of minutes to hours. We first introduce the innovation based on the Taylor expansion of the DWI, and later we introduce the MSR technique, which is employed in the model to further speed it up and making it memory efficient.

### 3.1. Taylor expansion based approximations

**Fig. 2.**
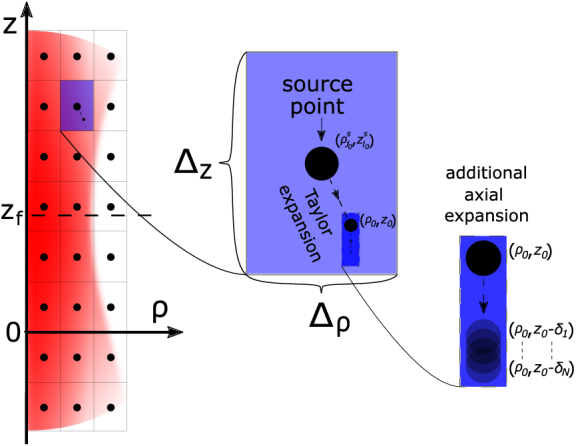
Grid scheme of the algorithm described in section 2). The source points $\left(\rho_{j}^{s},z_{l}^{s}\right)$ define rectangular sub-sets of the plane (ρ,z) . Given a point $\left(\rho_{0},z_{0}\right)$ belonging to the rectangular-neighborhood of $\left(\rho_{j_{0}}^{s},z_{l_{0}}^{s}\right)$ , the value of $h\left(\rho_{0},z_{0};k\right)$ is approximated by the Taylor expansion centered on $\left(\rho_{j_{0}}^{s},z_{l_{0}}^{s}\right)$ (see Eqs. (7a–7c)). The right side of the figure shows the additional axial expansion discussed in section 3.1.1). This further step is computed by setting $D_{z}^{0}>0$ in Eq. (9), which defines the algorithm calculating a second Taylor expansion centered at the point $\left(\rho_{0},z_{0}\right)$ , which is used to rapidly calculate many OCT simulations where scatterers are displaced in the axial direction (for example in the case of strain retrieval simulations). If $D_{z}^{0}=0$ , Eq. (9) coincides with the approximation of Eqs. (7), and no further axial expansion is performed.

In order to simplify notation, we consider only the y-component of Eq. (4), since it has fewer terms than the x-component, however the equivalent expression for the x-component immediately follows from the y-component expression:

(5) $$E_{y}^{inc}(r,z;k)=-ifk\sin(2\psi )\int_{0}^{\arcsin\left(\frac{\text{NA}}{\eta }\right)}g(\theta )e^{-{\left(\frac{k}{W}\right)}^{2}\sin^{2}(\theta )+i\eta kz\cos(\theta )}J_{2}\left(\eta k\rho \sin(\theta )\right)d\theta ,$$

where $g(\theta ):=\sqrt{\cos(\theta )}\sin(\theta )\left(\cos(\theta )-1\right)$ . Our goal is to calculate Eq. (5) in a fast and memory efficient way, for each point in space (r,z) and each wavenumber k . We achieve this by first evaluating Eq. (5) on a fixed sparse grid of relatively few points in the plane $\left(\rho_{j}^{s},z_{l}^{s}\right)$ which we denote as *source points*. We then use the Taylor expansion to calculate the incident field at arbitrary points within the plane (see Fig. (2)). We factor out axial phase oscillations present in the DWI by collecting the $e^{i\eta kz}$ term outside of the integral, since the Taylor expansion would otherwise be unsuitable to approximate the rapid phase oscillation of the DWI in the z direction:

(6a) $$E_{y}^{inc}(r,z;k)=-ifk\sin(2\psi )e^{i\eta kz}\cdot h\left(\rho ,z;k\right)$$

(6b) $$h\left(\rho ,z;k\right):=\int_{0}^{\arcsin\left(\frac{\text{NA}}{\eta }\right)}g(\theta )e^{-{\left(\frac{k}{W}\right)}^{2}\sin^{2}(\theta )+i\eta kz\left(\cos(\theta )-1\right)}J_{2}\left(\eta k\rho \sin(\theta )\right)d\theta .$$

This simple step of axial phase factorisation (Eq. (6a)) causes h(ρ,z;k) to become much less oscillatory than $E_{y}^{inc}(r,z;k)$ , making it suitable for expansion as a Taylor series. The main idea of the model is to numerically calculate the DWI, and its radial and axial derivatives up to a specified order, at only the source points, which are then employed to approximate the DWI at arbitrary points in space, as required to simulate each A-scan.

The grid of source points $\left(\rho_{j}^{s},z_{l}^{s}\right)$ is composed of $N_{\rho }$ and $N_{z}$ radial and axial coordinates, respectively, as shown in Fig. (2). The orders of the highest order derivatives are denoted by $D_{\rho }$ and $D_{z}$ , for the radial and axial derivatives, respectively. Equation (6b) is then approximated at each point ( $\rho_{0},z_{0}$ ) by evaluating the Taylor expansion of h , denoted $h_{T}$ , expanded about the closest source point of the source point grid $\left(\rho_{j_{0}}^{s},z_{l_{0}}^{s}\right)$ as:

(7a) $$h\left(\rho_{0},z_{0};k\right)\approx h_{T}\left(\rho_{0},z_{0};k\right):=\sum_{m=0}^{D_{\rho }}\sum_{n=0}^{D_{z}}\frac{{\left(\rho_{0}-\rho_{j_{0}}^{s}\right)}^{m}}{m!}\frac{{\left(z_{0}-z_{l_{0}}^{s}\right)}^{n}}{n!}S\left(m,n,j_{0},l_{0};k\right)$$

(7b) $$S\left(m,n,j,l;k\right):=\frac{\partial^{m+n}}{\partial \rho^{m}\partial z^{n}}{\left[h\left(\rho ,z;k\right)\right]}_{\left(\rho ,z)=(\rho_{j}^{s},z_{l}^{s}\right)}$$

(7c) $$j_{0}:=\underset{j=1:N_{\rho }}{argmin}\left|\rho_{0}-\rho_{j}^{s}\right|,\;l_{0}:=\underset{l=1:N_{z}}{argmin}\left|z_{0}-z_{l}^{s}\right|$$

The application of the Taylor expansion is justified by the fact that h(ρ,z;k) is an analytic function in both z and ρ variables [32]. Each derivative appearing in Eq. (7b) is calculated by analytically differentiating the inner function of Eq. (6b), and numerically evaluating the resulting integral, which is stored in the 5-D array S(m,n,j,l;k) , where m,n are indices related to the radial and axial derivative orders, j,l are the coordinates of the source point $\left(\rho_{j}^{s},z_{l}^{s}\right)$ and k is the wavenumber. Altogether, the algorithm calculates and stores $N_{TOT}=\left(D_{\rho }+1\right)\times \left(D_{z}+1\right)\times N_{\rho }\times N_{z}\times N_{k}$ integrals, where $N_{k}$ is the number of wavenumbers used in the simulation. In practice, the algorithm performs a calculation equivalent to the DWI itself $\left(D_{\rho }+1\right)\times \left(D_{z}+1\right)$ times, for the relatively small set of source points, which are then employed to generate each A-scan of the simulation. In section 4), we prove heuristically that an error which can be considered negligible for most applications, including phase-sensitive OCT simulations, is obtained by setting $D_{\rho }=20$ , $D_{z}=8$ , $s_{z}=190\mu$ m and $s_{\rho }=28\mu$ m, where $s_{z}$ and $s_{\rho }$ are the source point grid sampling rates in the axial and radial directions, respectively. As an example, when these parameters are chosen, $N_{\rho }=3$ and $N_{z}=6$ are sufficient to allow for accurate calculation of Eq. (7a) within a domain spanning 70μ m in the radial and 1 mm in the axial directions, respectively. $N_{k}$ determines the unambiguous OCT imaging depth, assuming a fixed system spectral width, and is chosen to satisfy the Nyquist Shannon sampling theorem, which guarantees that the FFT algorithm, which is needed to calculate a discrete version of Eq. (1b), can be employed to approximate the continuous Fourier transform. The number $N_{k}$ can be a bottleneck for simulations where scatterers are located deep in the sample, where $N_{k}$ can be two or three orders of magnitude larger than the other factors of $N_{TOT}$ . In section 3.2) we show how to overcome this obstacle to further speed up the simulation and dramatically reduce the memory usage, by calculating only a few monochromatic field components, which are used to calculate the field at any wavenumber in the spectrum. With these parameters, the numerical evaluation of the array S of integrals at the source points takes only few seconds.

**Fig. 3.**
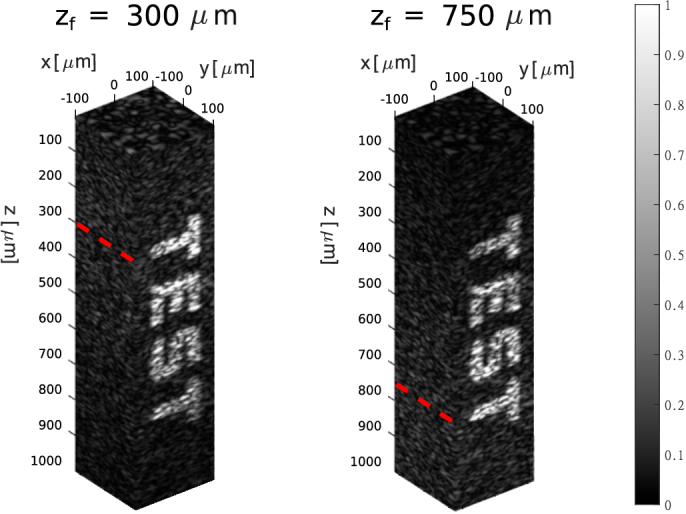
OCT C-scan simulations performed using the approximation based on the Taylor expansion. The two plots show the C-scan magnitude, normalized by dividing for its global maximum. Both plots share the same scatterer distribution, with varying focus points for each plot denoted as $z_{f}$ and highlighted by red dashed lines along the y axis.

Figure (3) shows an OCT C-scan simulated using the Taylor-based expansion. The 3D domain of scatterers spans 200μ m in both x and y directions, and the axial range is from 0 to 1 mm. The total number of randomly distributed scatterers in the region is $N_{s}=312500$ . This number was chosen to guarantee fully developed speckles, as reported by [33]. The number of wavenumbers is $N_{k}=865$ , a total of 200 of lateral scanning positions in x and y directions were used, resulting in a total of 40000 A-scans for each C-scan. The focus in the left and right plots is placed at $z_{f}=300\mu$ m and at $z_{f}=750\mu$ m, respectively, and was set by translating the z coordinate in the DWI. To demonstrate the capability of this model in simulating OCT signals from heterogeneous samples, we increased the cross-section $\rho_{s}$ of the scatterers within the "TEST" region to be five times larger than that of the remaining computational volume. As expected, in both cases the OCT signal is brighter near the focal region. The time required to calculate each C-scan was approximately 5 hours. The time required to calculate a single A-scan with the rigorous model was 7 minutes and 11 secs, thus meaning that it would take circa 200 days to rigorously calculate each C-scan. The rigorous generation of each C-scan of Fig. (3) requires the computation of $N_{k}\times N_{s}\times 200\times 200\approx 1.08\times 10^{13}$ numerical integrals. While the proposed method dramatically speeds up the computation, such large C-scans inherently require unfeasible time to compute.

#### 3.1.1 Additional expansion for axial displacements

We developed an additional expansion that further accelerates A-scan calculation when multiple OCT signals are calculated, each for a different set of microscopic scatterer axial displacements. This is particularly useful for simulating large OCE datasets, where the motion of the scatterers is assumed to be principally in the axial direction [7,26]. After having computed $h_{T}(\rho_{0},z_{0};k)$ , for each scatterer, the expansion explained in this section uses the same grid of source points $\left(\rho_{j}^{s},z_{l}^{s}\right)$ to calculate the Taylor expansion of its axial *derivatives* $\left(\frac{\partial^{n}}{\partial z_{0}^{n}}h_{T}(\rho_{0},z_{0};k)\right)$ , which is performed by analytically differentiating, with respect to $z_{0}$ , both sides of Eq. (7a). This process allows us to approximate the Taylor expansion of h in the z direction rapidly. In particular, we use the same array S to rapidly generate these new derivatives as:

(8) $$\begin{array}{rl} \frac{\partial^{n_{0}}}{\partial z^{n_{0}}}{\left[h_{T}\left(\rho_{0},z;k\right)\right]}_{z=z_{0}} & =\sum_{m=0}^{D_{\rho }}\sum_{n=n_{0}}^{D_{z}}\frac{{\left(\rho_{0}-\rho_{j_{0}}^{s}\right)}^{m}}{m!}\frac{{\left(z_{0}-z_{l_{0}}^{s}\right)}^{\left(n-n_{0}\right)}}{\left(n-n_{0}\right)!}S\left(m,n,j_{0},l_{0};k\right)\\ & =\sum_{m=0}^{D_{\rho }}\sum_{n=0}^{D_{z}-n_{0}}\frac{{\left(\rho_{0}-\rho_{j_{0}}^{s}\right)}^{m}}{m!}\frac{{\left(z_{0}-z_{l_{0}}^{s}\right)}^{n}}{n!}S\left(m,n+n_{0},j_{0},l_{0};k\right). \end{array}$$

We use Eq. (8) to approximate the the axial derivatives of h to generate a second Taylor expansion, centered on each scatterer $\left(\rho_{0},z_{0}\right)$ of the domain, as:

(9) $$h\left(\rho_{0},z_{0}+\delta ;k\right)\approx \sum_{n_{0}=0}^{D_{z}^{0}}\frac{\delta^{n_{0}}}{n_{0}!}\frac{\partial^{n_{0}}}{\partial z^{n_{0}}}{\left[h\left(\rho_{0},z;k\right)\right]}_{z=z_{0}}\approx \sum_{n_{0}=0}^{D_{z}^{0}}\frac{\delta^{n_{0}}}{n_{0}!}\frac{\partial^{n_{0}}}{\partial z^{n_{0}}}{\left[h_{T}\left(\rho_{0},z;k\right)\right]}_{z=z_{0}},$$

where $D_{z}^{0}$ is the number of additional axial derivatives. The second term of Eq. (9) is the Taylor polynomial of degree $D_{z}^{0}$ of $h\left(\rho_{0},z_{0};k\right)$ . In contrast, the third term, which constitutes the calculated value, is an approximation of the second term. In this approximation, each derivative is estimated using its respective Taylor expansion at the source points $\left(\rho_{j}^{s},z_{l}^{s}\right)$ , described by Eq. (8). Equations (8–9) can been used to rapidly simulate several OCT signals related to different configurations of the same sample, where the scatterers are microscopically displaced axially, in an arbitrary manner, for each configuration. This additional tool is particularly useful for generating OCT data underlying large OCE datasets, given its additional speed, which can be used for training deep learning strain retrieval models, for example.

As the degree of the Taylor polynomial on the right side of Eq. (8) decreases with respect to $n_{0}$ (where the maximum exponent of the terms $\left(z_{0}-z_{l_{0}}^{s}\right)$ is $D_{z}-n_{0}$ ), the accuracy of the corresponding approximation diminishes. To maintain consistent accuracy for all derivatives used in Eq. (9), it becomes necessary to calculate additional terms within the array S(m,n,j,l;k) . In particular, we need to calculate $D_{z}^{0}$ additional axial derivatives (for a total of $D_{z}+D_{z}^{0}$ ). The number of derivatives $D_{z}^{0}$ is chosen such that the error of the approximation in Eq. (9) is negligible, and this depends on the maximum axial scatterer displacement among all configurations of the sample. The expansion described in Eq. (9) is a generalisation of the basic Taylor expansion of Eq. (7), explained in the previous section. In fact, the latter is a specific case of Eq. (9), where $D_{z}^{0}=0$ .

### 3.2 Multi-spectral regression technique

In this section we describe a method that makes the computational time almost independent of the number of wavenumbers, allowing Eqs. (7–8) to be calculated directly for only a few wavenumbers, which are then used to evaluate these equations at all wavenumbers. To simplify the notation, let us rewrite h(ρ,z;k) as:

(10) $$h(\rho ,z;k)=\iint_{\Omega }d\left(s\right)e^{-a^{2}(s)k^{2}}e^{ib(s)k}ds_{x}ds_{y},$$

where a,b and d are functions of $s_{x},s_{y},\rho$ and z but do not depend on k . The equation above is found from Eq. (3) by setting $\phi (s_{x},s_{y};k)=e^{-{\left(\frac{k}{W}\right)}^{2}\left(s_{x}^{2}+s_{y}^{2}\right)}$ . In order to explain the MSR method, let us calculate Eq. (6b) for a subset of L wavenumbers $k_{1},k_{2},\ldots k_{L}$ . We look for L functions $C_{1}(k),C_{2}(k),\ldots ,C_{L}(k)$ that solve the following problem:

(11) $$e^{-\alpha^{2}k^{2}}e^{i\beta k}\approx \sum_{j=1}^{L}C_{j}(k)e^{-\alpha^{2}k_{j}^{2}}e^{i\beta k_{j}}\;\forall \alpha \in R,\;\beta \in [-B,B],\;k\in spectrum.$$

To find a suitable solution of Eq. (11), we define an integral least squares minimisation problem that we solve by applying the gradient descent method [34] (see section 3) of the Supplement 1 for more details). Once the functions $C_{1}(k),C_{2}(k),\ldots ,C_{L}(k)$ have been found, we can use Eqs. (10) and (11) to approximate h(ρ,z;k) , for *an arbitrary* k , as a linear combination of $h(\rho ,z;k_{1}),..,h(\rho ,z;k_{L})$ as follows (see section 1) of the Supplement 1 for more details):

(12a) $$h(\rho ,z;k)=\iint_{\Omega }d\left(s\right)e^{-a^{2}(s)k^{2}}e^{ib(s)k}ds_{x}ds_{y}=e^{ik\eta \Delta }\iint_{\Omega }d\left(s\right)e^{-a^{2}(s)k^{2}}e^{i\left(b(s)-\eta \Delta \right)k}ds_{x}ds_{y}\;$$

(12b) $$\approx \sum_{j=1}^{L}C_{j}(k)e^{i\left(k-k_{j}\right)\eta \Delta }h(\rho ,z;k_{j}),$$

where Δ is an arbitrary term such that |b(s)−ηΔ|<B . Namely, the right hand side of Eq. (12) is approximately constant as a function of Δ . By substituting Eq. (12) into Eq. (6a) for each component of the electric field, we can evaluate the DWI as:

(13) $$E(r,z;k)\approx \sum_{j=1}^{L}\frac{k}{k_{j}}C_{j}(k)e^{i\eta (k-k_{j})(z+\Delta )}E(r,z;k_{j}),$$

We would like to substitute Eq. (13) into Eq. (2a) and find a way to calculate the $\alpha_{scat}(k)$ as a function of $\alpha_{scat}(k_{j})$ , so as to make the computation of Eq. (13) independent of the number of scatterers, which would dramatically reduce the computation cost of calculating $\alpha_{scat}(k)$ . This goal is made difficult by the presence of the phase term $\left(e^{i\eta \left(k-k_{j}\right)\left(z+\Delta \right)}\right)$ in Eq. (13), which depends on the scatterer axial coordinate z . To overcome this obstacle, we first divide the region containing scatterers into depth bands of thickness D , each centered on the plane $z={\hat{z}}_{h}$ , and then we consider Eq. (13) with $\Delta ={\hat{z}}_{h}-z$ to replace $\left(e^{i\eta \left(k-k_{j}\right)\left(z+\Delta \right)}\right)$ with $\left(e^{i\eta \left(k-k_{j}\right){\hat{z}}_{h}}\right)$ , for each scatterer in the same band. This step transfers the computationally expensive dependence of Eq. (13) on single scatterers to depth bands, and we show how this is useful to consistently reduce the RAM usage and the computational time. Let us call $I_{h}$ the subset of indices ( s ) of the scatterers $P_{s}:=\left(x_{s},y_{s},z_{s}\right)$ , belonging to the h -th band $I_{h}:=\left\{s\in N|\left|z_{s}-{\hat{z}}_{h}\right|\leq \frac{D}{2},s\leq N_{s}\right\}$ . We denote by $z_{s_{h}}$ the axial coordinates $z_{s}$ of the scatterers having index $s\in I_{h}$ . By substituting Eq. (13) into Eq. (2a), and choosing $\Delta ={\hat{z}}_{h}-z_{s_{h}}$ for each $s_{h}\in I_{h}$ , we find (see section 2 of the Supplement 1 for more details):

(14) $$\alpha_{scat}\left(k\right)\approx \sum_{h=1}^{H}e^{i\eta 2k{\hat{z}}_{h}}\sum_{j_{1}=1}^{L}\sum_{j_{2}=1}^{L}\frac{k^{2}}{k_{j_{1}}k_{j_{2}}}C_{j_{1}}(k)C_{j_{2}}(k)e^{-i\eta (k_{j_{1}}+k_{j_{2}}){\hat{z}}_{h}}\alpha_{cross}(k_{j_{1}},k_{j_{2}},h)$$

where $\alpha_{cross}(k_{j_{1}},k_{j_{2}},h):=\sum_{s_{h}\in I_{h}}\rho_{s_{h}}E_{x}^{inc}\left(P_{s_{h}};k_{j_{1}}\right)E_{x}^{inc}\left(P_{s_{h}};k_{j_{2}}\right)$ is called *cross-modal coefficient*. The computational load of Eq. (14) is mainly related to the calculation of the terms $\alpha_{cross}(k_{j_{1}},k_{j_{2}},h)$ , which is not demanding because each term considers a small number of the scatterers ( $I_{h}$ subregions).

## 4. Analysis of accuracy and computational time

**Fig. 4.**
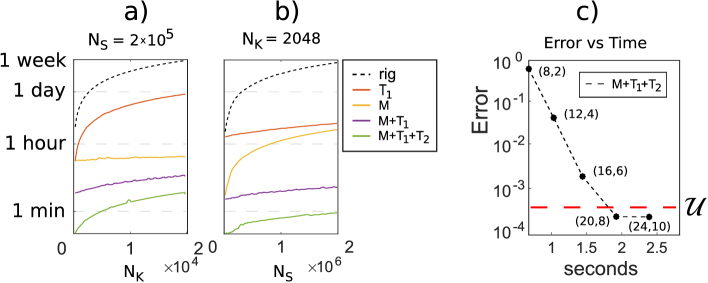
Plots a) and b) show computational times required to calculate one unloaded and 50 loaded A-scans on a log scale, by using the three expansions separately and together, and each data point forming each curve is a complete simulation. In a) we increased the number of wavenumbers $N_{k}$ , in b) we increased the number of scatterers $N_{s}$ . Plot c) computational time versus integral error of the simulation of an OCT A-scan by using the approximation. In order to test the Taylor expansion $T_{2}$ , a distribution of scatterers $z_{s_{j}}$ was initially generated, and a high strain $\hat{\epsilon }$ was applied to introduce an axial displacement $z_{s_{j}}^{L}:=z_{s_{j}}(1+\hat{\epsilon })$ . Later, the OCT A-scan related to $z_{s_{j}}^{L}$ was generated from the signal of $z_{s_{j}}$ , according to Eq. (9).

We have introduced three approximations in this paper, including the two Taylor approximations and the MSR. It is important to note that all of these are integrated into a single model, which is used to generate the results presented in this section. The only exception to this is the results shown in Figs. (4(a)) and (4(b)), where we compare the different approximations. In this section we analyze the computational time of the presented model, and we illustrate its accuracy by calculating a relative integral error. As explained in section 2), we denote by *rigorous* each OCT simulation evaluated using Eqs. (1–2), where the incident field has been calculated with the DWI without any approximation, and by *approximate* each simulation made with the new model, namely, by using Eq. (13) where each component of $E(r,z;k_{j})$ is calculated with Eq. (9). To quantify the accuracy of each approximation, we have used the following integral error:

(15) $$Err(F^{a}):=\sqrt{\frac{\int_{S}{\left|\left|F^{r}(P)-F^{a}(P)\right|\right|}^{2}dP}{\int_{S}{\left|\left|F^{r}(P)\right|\right|}^{2}dP}},$$

where $F^{r}$ and $F^{a}$ are electric fields or OCT signals simulated using the rigorous and approximate method, respectively. In the case of OCT signals, the data can be A-scans, B-scans or C-scans. The variable P and the integration domain S can be 1D, 2D, or 3D, depending on the arrays being compared. For example, 1D for A-scans, 2D for B-scans and 3D for C-scans.

It is worth noting that each of the three approximations can be calculated with arbitrary accuracy, at the cost of computational time. Thus, we need to trade computation time for precision. In order to analyze computational time and accuracy we set an upper bound U for the integral error, namely, we consider negligible any error smaller than U . To choose U , we simulated several OCT and data sets, for the rigorous and approximate cases, and we observed that approximate simulations $F^{a}$ for which $Err(F^{a})\leq 10^{-3}$ , produced results nearly indistinguishable from those obtained through rigorous simulations ( $F^{a}\approx F^{r}$ ). In view of this we chose a conservative value of $U=3\times 10^{-4}$ .

### 4.1 Parameter setting

Accuracy and computational time depend upon the parameters employed in the simulation, which we divide into physical parameters and approximation parameters. To facilitate the calculations presented in this section, we choose practically relevant values for the physical parameters. These include: NA=0.1 , $\rho_{max}=70\mu$ m, $z_{max}=1$ mm, η=1 , where $\rho_{max}$ and $z_{max}$ are the maximum distance of the scatterers from the optical axis (distant scatterers can be considered negligible for the DWI) and the axial coordinate of the deepest scatterer, respectively. We set the spectrum to [1170,1408] nm to approximately match that of our Thorlabs TELESTO-II - spectral domain OCT system. The setting of the approximation parameters can be performed independently for each approximation. The parameters which must be set for the Taylor expansion are $D_{\rho },D_{z}$ and $s_{\rho },s_{z}$ , while and $N_{z},N_{\rho }$ are easily found as a function of their related sampling rates. For each choice of the first two, we can find values for the others such that the approximation error is smaller than U . By running several simulations we empirically found that $D_{\rho }=20,D_{z}=8$ is an optimal solution for the computational time, to which correspond sampling rates $s_{\rho }=28\mu$ m $,s_{z}=190\mu$ m $,N_{z}=6$ and $N_{\rho }=3$ . The MSR parameters are L,T,D and B . In a similar way to the Taylor expansion, by setting L and T we can find D to satisfy the threshold U . Empirically we found L=35,T=4 ,which corresponds to D=15μ m. A suitable value for B is given by (see section 4) of the Supplement 1 for more details):

(16) $$B=\rho_{max}NA+z_{max}\frac{NA^{2}}{\eta }+\eta \frac{D}{2}.$$

By replacing the chosen physical parameters and D in Eq (16) we find B=25μ m. The parameter employed in the additional Taylor expansion is $D_{z}^{0}$ , which is non-zero only if axial scatterer motion is simulated, in which case multiple distributions of scatterers are generated. In the case of simulation of $N_{d}$ axial displacements, let us call $\{z_{j}(d){\}}_{d=1:N_{d}}$ all the subsequent axial coordinates of the $j^{th}$ scatterer. We heuristically found that $D_{z}^{0}:=\lceil 0.15\times M_{s}\rceil$ maintains the error below U , where ⌈⋅⌉ is the ceiling function and $M_{s}:=\max_{j=1:N_{s}}\left\{\max_{d=1:N_{d}}\left\{z_{j}(d)\right\}-\min_{d=1:N_{d}}\left\{z_{j}(d)\right\}\right\}$ is the maximum axial displacement among all scatterers measured in μ m.

### 4.2 Comparison of the three approximations

Figures (4(a)) and (4(b)) compare the computational time of the three approximations described, i.e., the two Taylor expansions and the MSR, which are labeled as $T_{1}$ , $T_{2}$ , and M, respectively. Each data point of each plotted curve represents the result of an entire simulation, consisting in the generation of one unloaded and 50 loaded A-scans ( $N_{d}=51$ ). In Fig. (4(a)), we simulated $N_{s}=2\times 10^{5}$ scatterers, while the number of wavenumbers $N_{k}$ varied from $10^{2}$ to $2\times 10^{4}$ . In Fig. (4(b)), each simulation employed $N_{k}=2048$ wavenumbers, while the number of scatterers $N_{s}$ varied from $10^{2}$ to $2\times 10^{6}$ . In both plots, the improvement due to MSR is higher than that due to the Taylor expansions, because we chose extremely challenging simulations, which employ a large value of $N_{k}$ , where MSR becomes vital. The use of all approximations proves to be much more efficient than each individually. In particular, MSR makes the model particularly suitable for swept-source OCT image formation simulations, where a large number of wavenumbers are often needed. Finally, the inclusion of the additional Taylor expansion makes a further reduction in computational time, by a factor of 3 to 10 in both plots. Fig. (4(c)) shows a plot of computational time versus accuracy for an OCT A-scan simulated using the three approximations. In order to use the additional Taylor expansion, we initially generated an A-scan (unloaded), and then the axial coordinate $z_{j}$ of each scatterer was displaced introducing a large strain $\hat{\epsilon }=-10m\epsilon$ , and a loaded A-scan was calculated from the unloaded A-scan, by employing the additional Taylor expansion (see Eq. (9)). We considered 64000 scatterers having axial coordinates between 0.5 mm and 1 mm, deliberately choosing a deep axial region to challenge the algorithm. Each of the 5 points in the plot represents a computation of the loaded A-scan, where $D_{z}^{0}=2$ , as calculated using the formula provided in section 4.1. In each simulation, we systematically increased the values of $D_{\rho }$ and $D_{z}$ . As expected, at each step, the computational time increased and the error decreased. The red dashed line represents the upper bound U . Notably, the error in the last two simulations was smaller than U , showing consistency with the parameters setting that we described in the previous section. The analysis presented in this section validates that the model, utilizing all three approximations and run with $D_{\rho }=20$ and $D_{z}=8$ achieves the optimal computational time while maintaining optimal precision, as depicted in the Fig. (4). Based on these results, we recommend this model configuration for *all* simulations.

### 4.3 Rapid simulation of multiple C-scans

**Fig. 5.**
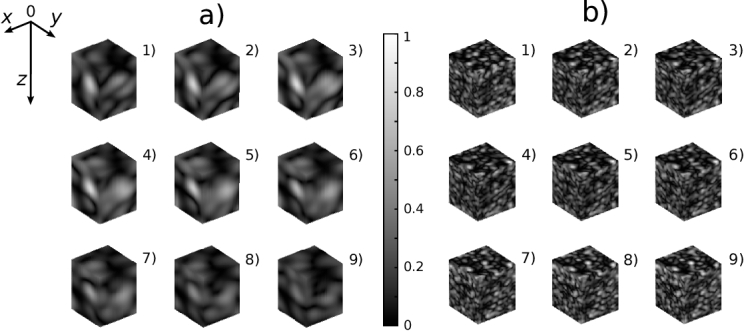
Two examples of OCT C-scan dataset generation. a) and b) show OCT C-scans with volumes of 20μ m ×20μ m ×25μ m and 80μ m ×80μ m ×90μ m, respectively. Each simulation includes 1000 C-scans and uniform axial strains ranging from $10^{-2}m\epsilon$ to 10mϵ . Each figure shows the OCT magnitude normalised by the same factor. The plots are arranged in order of increasing strain, with plot 1 and plot 9 having the lowest and the highest strains, respectively.

Figure (5) shows two examples of simulation of a large number of OCT C-scans, which have been rapidly simulated, to demonstrate the model speed. Both examples involve the simulation of 1000 OCT C-scans at different strain levels, of which 9 are shown. The uniform axial strain is incrementally increased in steps of $10^{-2}m\epsilon$ , reaching a maximum of 10mϵ . In Fig. (5(a)), each C-scan is composed of 29×29 A-scans in a volume of 20μ m ×20μ m ×25μ m with 1280 scatterers. The computational time was 27 minutes and 3 seconds, compared with an estimated computation time for the rigorous method of 39 hours. The plots in Fig. 5(b)) are for a larger field of view of 48×48 A-scans per C-scan, representing a volume of 80μ m ×80μ m ×90μ m containing 7200 scatterers. The computation time was 6 hours and 53 minutes, and in this example, we have estimated more than 114 days would be required in the case of rigorous calculation using DWI. An example of application of these datasets is the training dataset generation for a DL model for strain retrieval in OCE that predicts the strain for each pixel by taking in input small volumes of unloaded and loaded OCT C-scans related to the spatial region surrounding the pixel, and giving in output the related strain, which is assumed locally uniform. This assumption of uniform strain is not restrictive, and is taken by conventional models in OCE, including WLS [10] and VM [35].

### 4.4 Example of strain retrieval in OCE from simulated OCT raw data

Now we compare the rigorous and approximate methods for generating unloaded and loaded OCT signals that are used as input for the weighted least square method (WLS) for strain retrieval in OCE, with a similar approach to the one described by Kennedy *et al.*[10]. The following steps were conducted twice, once for the rigorous approach and once for the approximate method. We simulated a 3D computational volume of size 1000μ m ×140μ m ×500μ m in the x,y and z directions, respectively. Our primary objective was to obtain a 2D elastogram at the plane y=70μ m. However, due to the optimal performance of the WLS method’s unwrapping algorithm with 3D OCE data, OCT C-scans comprising seven unloaded/loaded pairs of B-scans were calculated at adjacent planes, namely y=70μ m $+\Delta_{y}$ , where $\Delta_{y}=[-3.6,-2.4,-1.2,0,1.2,2.4,3.6]\mu$ m. For each B-scan, 825 A-scans were calculated, with a lateral scanning step of 1.2μ m. We used a simple mechanical model based on a stiff inclusion in the form of an elliptic cylinder. Specifically, the cross-section of the inclusion at the plane y=0 is elliptical, and it extends uniformly throughout the entire computational domain in the y -direction. To simulate mechanical loading, we set ${\hat{\epsilon }}_{1}=-0.5m\epsilon$ inside the inclusion, while ${\hat{\epsilon }}_{2}=-3m\epsilon$ was applied to the remaining regions within the computational volume.

**Fig. 6.**
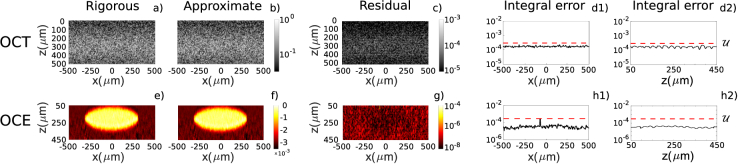
Comparison of simulations of pairs unloaded/loaded OCT signals and related strains retrieved with the WLS method, for the rigorous and the approximate cases. a-b) Normalized magnitudes of the OCT B-scan on a log-scale, for the rigorous and approximate case, respectively. c) Absolute value of the residual image of the OCT B-scans shown in a) and b). g) Absolute value of the residual image of e) and f). d1-d2) integral error of the central B-scan along the axial and lateral direction, plotted as a function of the lateral and axial direction, in h1) and h2), respectively. h1-h2) integral error of the strains along the axial and lateral direction, plotted as a function of the lateral and axial direction, in h1) and h2), respectively.

Lastly, the WLS method was used with the pairs of generated OCT C-scans to retrieve the strain distribution, and the central elastogram ( y=70μ m) was extracted and compared in Fig. (6). Figs. (6(a)) and (6(b)) show the central unloaded B-scan magnitude on a log scale, normalized by its maximum magnitude, for the rigorous and approximate cases, respectively. Fig. (6(c)) illustrates the absolute value of the difference between the complex-valued rigorous and approximate phase-sensitive OCT calculations, within the dynamic range of $\left[10^{-5},10^{-3}\right]$ to enhance visibility. Fig. (6(d1)) and Fig. (6(d2)) illustrate the integral error of the two B-scans shown in Figs. (6(a-b)), evaluated for each OCT A-scan and plotted as function of x and z , respectively.

Figure (6(e)) and (6(f)) show the retrieved elastograms, for the accurate and the approximated methods, respectively. It is important to highlight that while Fig. (6(e)) represents the rigorous case, the ellipse is slightly blurred since it represents the retrieved strain, in contrast to the image of the strain map, which has sharp boundaries between regions of differing strain. Fig. (6(g)) illustrates the absolute value of their difference within a dynamic range of $\left[10^{-8},10^{-4}\right]$ , chosen to enhance visibility. Fig. (6(h1)) and Fig. (6(h2)) show the integral error of the two elastograms shown in Fig. (6(e))-(6(f)) along the axial and x direction, plotted as function of x and z , respectively. The dashed red lines in the plots d1), d2), h1) and h2) of Fig. (6) indicate the threshold U . The integral error in this case is even smaller than in the OCT case. This outcome is expected since, for each pixel, the strain is estimated by performing linear regression on an axial range surrounding that pixel in the respective OCT image. While this process reduces the resolution of OCE images compared to their OCT counterparts, it effectively averages and diminishes the error introduced by the approximation.

Each simulation presented in this article was computed using Matlab’s (Matlab R2021b) with an NVIDIA A6000 48GB GPU, and two CPU AMD EPYC 7453 with 28-Cores.

## 5. Conclusion

We have presented a fast, efficient and flexible model to simulate OCT image formation, which relies on the first-order Born approximation for the calculation of the scattered field, without making the paraxial approximation. The model allows arbitrary scatterer distributions in the focal region, arbitrary lateral sample scanning, simulations of aberrations and customizable spectral bandwidth. We showed that the model rapidly calculates OCT A-scans by efficiently combining the Taylor expansion formula and the MSR technique, that we have introduced, and which can be used to approximate arbitrary monochromatic components with high accuracy. MSR is largely employed in the model to dramatically reduce the memory usage and further speed it up, allowing simulations with large number of scatterers, wavenumbers and lateral scannings.

The model has an additional sub-algorithm that makes it 3-10 times faster in simulating series of OCT C-scans, where the scatterers are arbitrarily (not necessarily uniformly) axially shifted. If lateral strains are needed, the model can be used in conjunction with more general mechanical loading simulators, such as finite-element-methods, at the cost of losing this additional speed. The model is suitable for generating large datasets of OCT signals, which can be potentially used for training deep learning models that use OCT raw data. Furthermore, the growth of the computation time is negligible as a function of the number of wavenumbers, making it robust in simulating deep OCT C-scans. We showed a comparison of the three approximations of the model, clarifying their importance. We analysed the error and computational time, to demonstrate the validity and the accuracy of the model, including an example of strain retrieval in OCE.

## Supplemental information

Supplement 1 Supplemental document https://doi.org/10.6084/m9.figshare.27331239

## Data Availability

Data underlying the results presented in this paper are available in Ref. [36].

## Supplemental document

See Supplement 1 for supporting content.

## References

[1] Izatt J. A. Choma M. A. Dhalla A.-H., “Theory of optical coherence tomography,” in *Optical Coherence Tomography: Technology and Applications* , 6th Edition, vol. 1, W. Drexler Fujimoto J. G., eds. (Springer International Publishing Switzerland, 2015), chap. 2, pp. 65–94, 2nd ed.

[2] Kishi S., “Impact of swept source optical coherence tomography on ophthalmology,” Taiwan Journal of Ophthalmology 6(2), 58–68 (2016). 10.1016/j.tjo.2015.09.002 29018713 PMC5602691

[3] Olsen J. Holmes J. Jemec G. B. E., “Advances in optical coherence tomography in dermatology-a review,” J. Biomed. Opt. 23(04), 040901 (2018). 10.1117/1.JBO.23.4.040901 29701018

[4] Yonetsu T. Bouma B. E. Kato K. et al., “Optical coherence tomography: 15 years in cardiology,” Circ. J. 77(8), 1933–1940 (2013). 10.1253/circj.CJ-13-0643.1 23856651

[5] Tsai T.-H. Lee H.-C. Ahsen O. O. et al., “Ultrahigh speed endoscopic optical coherence tomography for gastroenterology,” Biomed. Opt. Express 5(12), 4387–4404 (2014). 10.1364/BOE.5.004387 25574446 PMC4285613

[6] Kennedy B. F. Wijesinghe P. Sampson D. D., “The emergence of optical elastography in biomedicine,” Nat. Photonics 11(4), 215–221 (2017). 10.1038/nphoton.2017.6

[7] Kennedy B. F. Kennedy K. M. Sampson D. D., “A review of optical coherence elastography: Fundamentals, techniques and prospects,” IEEE J. Select. Topics Quantum Electron. 20(2), 272–288 (2014). 10.1109/JSTQE.2013.2291445

[8] Matveyev A. L. Matveev L. A. Moiseev A. A. et al., “Computationally efficient model of oct scan formation by focused beams and its usage to demonstrate a novel principle of OCT-angiography,” Laser Phys. Lett. 17(11), 115604 (2020). 10.1088/1612-202X/abac16

[9] Schmitt J. M. Knüttel A., “Model of optical coherence tomography of heterogeneous tissue,” J. Opt. Soc. Am. A 14(6), 1231–1242 (1997). 10.1364/JOSAA.14.001231

[10] Chin L. Curatolo A. Kennedy B. F. et al., “Analysis of image formation in optical coherence elastography using a multiphysics approach,” Biomed. Opt. Express 5(9), 2913–2930 (2014). 10.1364/BOE.5.002913 25401007 PMC4230875

[11] Yao G. Wang L. V., “Monte Carlo simulation of an optical coherence tomography signal in homogeneous turbid media,” Phys. Med. Biol. 44(9), 2307–2320 (1999). 10.1088/0031-9155/44/9/316 10495123

[12] Tycho A. Jørgensen T. M. Yura H. T. et al., “Derivation of a Monte Carlo method for modeling heterodyne detection in optical coherence tomography systems,” Appl. Opt. 41(31), 6676–6691 (2002). 10.1364/AO.41.006676 12412659

[13] Kirillin M. Meglinski I. Kuzmin V. et al., “Simulation of optical coherence tomography images by Monte Carlo modeling based on polarization vector approach,” Opt. Express 18(21), 21714–21724 (2010). 10.1364/OE.18.021714 20941071

[14] Wang Y. Bai L., “Accurate Monte Carlo simulation of frequency-domain optical coherence tomography,” Numer. Methods Biomed. Eng. 35(4), 19 (2019). 10.1002/cnm.3177 PMC6492136 30690893

[15] Munro P. R. T. Curatolo A. Sampson D. D., “Full wave model of image formation in optical coherence tomography applicable to general samples,” Opt. Express 23(3), 2541–2556 (2015). 10.1364/OE.23.002541 25836119

[16] Munro P. R. T., “Three-dimensional full wave model of image formation in optical coherence tomography,” Opt. Express 24(23), 27016–27031 (2016). 10.1364/OE.24.027016 27857429

[17] Macdonald C. M. Munro P. R. T., “Approximate image synthesis in optical coherence tomography,” Biomed. Opt. Express 12(6), 3323–3337 (2021). 10.1364/BOE.420992 34221663 PMC8221936

[18] Munro P. R. T., “Tool for simulating the focusing of arbitrary vector beams in free-space and stratified media,” J. Biomed. Opt. 23(09), 040901 (2018). 10.1117/1.JBO.23.9.090801 30251490

[19] Matveyev A. L. Matveev L. A. Moiseev A. A. et al., “Semi-analytical full-wave model for simulations of scans in optical coherence tomography with accounting for beam focusing and the motion of scatterers,” Laser Phys. Lett. 16(8), 085601 (2019). 10.1088/1612-202X/ab2243

[20] Matveyev A. L. Matveev L. A. Moiseev A. A. et al., “Simulating scan formation in multimodal optical coherence tomography: angular-spectrum formulation based on ballistic scattering of arbitrary-form beams,” Biomed. Opt. Express 12(12), 7599–7615 (2021). 10.1364/BOE.440739 35003855 PMC8713662

[21] Zykov A. A. Matveyev A. L. Matveev L. A. et al., “Flexible computationally efficient platform for simulating scan formation in optical coherence tomography with accounting for arbitrary motions of scatterers,” Journal of Biomedical Photonics and Engineering 7(1), 010304 (2021). 10.18287/JBPE21.07.010304

[22] Brenner T. Reitzle D. Kienle A., “Optical coherence tomography images simulated with an analytical solution of Maxwell’s equations for cylinder scattering,” J. Biomed. Opt. 21(4), 045001 (2016). 10.1117/1.JBO.21.4.045001 27032336

[23] Brenner T. Munro P. R. T. Krüger B. et al., “Two-dimensional simulation of optical coherence tomography images,” Sci. Rep. 9(1), 12189 (2019). 10.1038/s41598-019-48498-2 31434928 PMC6704163

[24] Richards B. Wolf E., “Electromagnetic diffraction in optical systems, II. Structure of the image field in an aplanatic system,” Proc. R. Soc. Lond. A 253(1274), 358–379 (1959). 10.1098/rspa.1959.0200

[25] Wolf E., “Electromagnetic diffraction in optical systems - I. An integral representation of the image field,” Proc. R. Soc. Lond. A 253, 349–357 (1959). 10.1098/rspa.1959.0199

[26] Zaitsev V. Y. Matveyev A. L. Matveev L. A. et al., “Strain and elasticity imaging in compression optical coherence elastography: The two-decade perspective and recent advances,” J. Biophotonics 14(2), 32 (2021). 10.1002/jbio.202000257 32749033

[27] Ossowski P. Curatolo A. Sampson D. D. et al., “Realistic simulation and experiment reveals the importance of scatterer microstructure in optical coherence tomography image formation,” Biomed. Opt. Express 9(7), 3122–3136 (2018). 10.1364/BOE.9.003122 29984087 PMC6033572

[28] Munro P. R. T., “Exploiting data redundancy in computational optical imaging,” Opt. Express 23(24), 30603–30617 (2015). 10.1364/OE.23.030603 26698693

[29] Born M. Wolf E., “Diffraction by a conducting sphere; theory of Mie,” in *Principles of Optics* , Cambridge University Press, ed. (Cambridge University Press, 1959), chap. 14.5, pp. 759–789, seventh ed.

[30] Wyld H. W., “Bessel Functions and Applications,” *Mathematical Methods for Physics* , (CRC Press, 2019), chap. 4, pp. 190–192.

[31] Lüneburg R. K., “Diffraction of converging spherical waves,” *Mathematical Theory of Optics* , (University of California Press, 1964), chap. 46, pp. 321–324.

[32] Lars V. Ahlfors, “Cauchy’s Integral formula,” in *Complex Analysis: An Introduction to the Theory of Analytic Functions of One Complex Variable* , (McGraw-Hill, 1979), chap. 4, pp. 114–120, 3rd ed.

[33] Hillman T. R. Adie S. G. Seemann V. et al., “Correlation of static speckle with sample properties in optical coherence tomography,” Opt. Lett. 31(2), 190–192 (2006). 10.1364/OL.31.000190 16441026

[34] Bin S. Iyengar S. S., “Optimization Formulation,” in *Mathematical Theories of Machine Learning - Theory and Applications* , (Springer Nature, 2020), chap. 3, pp. 17–27.

[35] Matveyev A. L. Matveev L. A. Sovetsky A. A. et al., “Vector method for strain estimation in phase-sensitive optical coherence elastography,” Laser Phys. Lett. 15(6), 065603 (2018). 10.1088/1612-202X/aab5e9

[36] Mazzolani A., “Data supporting a fast and customizable image formation model for optical coherence tomography,” Zenodo 2024, 10.5281/zenodo.12594466. PMC11640576 39679414

