# Supplementary material for: Fast and customizable image formation model for optical coherence tomography
Source: Biomed Opt Express. 2024 Nov 13;15(12):6783–98. doi: 10.1364/BOE.534263 (PMC11640576; doi:10.1364/BOE.534263)
Supplement: Supplementary file 1 [file boe-15-12-6783-s001.pdf]

Supplemental Document

Biomedical Optics EXPRESS

Fast and customizable image formation model for optical coherence tomography: supplement

Andrea Mazzolani,* Callum Macdonald, and Peter R. T. Munro

Department of Medical Physics and Biomedical Engineering, University College London, Malet Place, Gower Street, London WC1E 6BT, UK

*andrea.mazzolani.18@ucl.ac.uk

This supplement published with Optica Publishing Group on 13 November 2024 by The Authors under the terms of the Creative Commons Attribution 4.0 License in the format provided by the authors and unedited. Further distribution of this work must maintain attribution to the author(s) and the published article's title, journal citation, and DOI.

Supplement DOI: https://doi.org/10.6084/m9.figshare.27331239

Parent Article DOI: https://doi.org/10.1364/BOE.534263

A fast and customizable image formation model for optical coherence tomography (Supplemental document)

1. MULTI-SPECTRAL REGRESSION (MSR)

In this section we demonstrate Eq.(12) of the primary document. For each Δ s.t. the β-upper bound (B) of Eqs.(11) of the primary document is not exceeded, namely, $\forall\Delta$ s.t. $|b(s) - \eta\Delta| < B$, we have:

$$
\begin{aligned}
h(\rho, z; k) &= \iint_{\Omega} d\left(\boldsymbol{s}\right) e^{-a^2(\boldsymbol{s})k^2} e^{ib(\boldsymbol{s})k} ds_x ds_y \\
&= \left(e^{ik\eta\Delta} e^{-ik\eta\Delta}\right) \iint_{\Omega} d\left(\boldsymbol{s}\right) e^{-a^2(\boldsymbol{s})k^2} e^{ib(\boldsymbol{s})k} ds_x ds_y \\
&= e^{ik\eta\Delta} \iint_{\Omega} d\left(\boldsymbol{s}\right) e^{-a^2(\boldsymbol{s})k^2} e^{i\left(b(\boldsymbol{s})-\eta\Delta\right)k} ds_x ds_y \\
&\approx e^{ik\eta\Delta} \iint_{\Omega} d\left(\boldsymbol{s}\right) \sum_{j=1}^{L} \mathcal{C}_j(k) e^{-a^2(\boldsymbol{s})k_j^2} e^{i\left(b(\boldsymbol{s})-\eta\Delta\right)k_j} ds_x ds_y \\
&= e^{ik\eta\Delta} \sum_{j=1}^{L} \mathcal{C}_j(k) \iint_{\Omega} d\left(\boldsymbol{s}\right) e^{-a^2(\boldsymbol{s})k_j^2} e^{i\left(b(\boldsymbol{s})-\eta\Delta\right)k_j} ds_x ds_y \\
&= \sum_{j=1}^{L} \mathcal{C}_j(k) e^{i\left(k-k_j\right)\eta\Delta} \iint_{\Omega} d\left(\boldsymbol{s}\right) e^{-a^2(\boldsymbol{s})k_j^2} e^{ib(\boldsymbol{s})k_j} ds_x ds_y \\
&= \sum_{j=1}^{L} \mathcal{C}_j(k) e^{i\left(k-k_j\right)\eta\Delta} h(\rho, z; k_j)
\end{aligned}
$$

2. MODAL COEFFICIENTS APPROXIMATION BY EMPLOYING MSR

In this section we demonstrate Eq.(14) of the primary manuscript. Let us call I_h the subset of indices (s) of the scatterers ($\boldsymbol{P}_s := (x_s, y_s, z_s)$) belonging to the h-th band $\left(I_h := \left\{s \in \mathbb{N} \middle| \, |z_s - \hat{z}_h| \leq \frac{D}{2}\right\}\right)$. We denote z_{s_h} the axial coordinates z_s such that $s \in I_h$. Then, by replacing Eq.(13) in Eq.(2a) of the

primary document, and choosing $\Delta = \hat{z}_h - z_{s_h}$ for each $s_h \in I_h$, we find:

$$\begin{aligned}
\alpha_{scat}(k) &= \sum_{s=1}^{N_s} \rho_s \left(E_x^{inc}(\boldsymbol{P}_s; k) \right)^2 \\
&\approx \sum_{s=1}^{N_s} \rho_s \left(\sum_{j=1}^{L} \frac{k}{k_j} \mathcal{C}_j(k) e^{i\eta(k-k_j)(z_s+\Delta)} \boldsymbol{E}(\boldsymbol{r}, z_s; k_j) \right)^2 \\
&= \sum_{h=1}^{H} \sum_{s_h \in I_h} \rho_{s_h} \left(\sum_{j=1}^{L} \frac{k}{k_j} \mathcal{C}_j(k) e^{i\eta(k-k_j)(z_{s_h}+\Delta)} \boldsymbol{E}(\boldsymbol{r}, z_{s_h}; k_j) \right)^2 \\
&\approx \sum_{h=1}^{H} \sum_{s_h \in I_h} \rho_{s_h} \left(\sum_{j=1}^{L} \frac{k}{k_j} \mathcal{C}_j(k) e^{i\eta(k-k_j)\hat{z}_h} E_x^{inc}\left(\boldsymbol{P}_{s_h}; k_j\right) \right)^2 \\
&= \sum_{h=1}^{H} \sum_{s_h \in I_h} \rho_{s_h} \sum_{j_1=1}^{L} \sum_{j_2=1}^{L} \frac{k^2}{k_{j_1} k_{j_2}} \mathcal{C}_{j_1}(k) \mathcal{C}_{j_2}(k) e^{i\eta(k-k_{j_1})\hat{z}_h} e^{i\eta(k-k_{j_2})\hat{z}_h} E_x^{inc}\left(\boldsymbol{P}_{s_h}; k_{j_1}\right) E_x^{inc}\left(\boldsymbol{P}_{s_h}; k_{j_2}\right) \\
&= \sum_{h=1}^{H} e^{i\eta 2k\hat{z}_h} \sum_{j_1=1}^{L} \sum_{j_2=1}^{L} \frac{k^2}{k_{j_1} k_{j_2}} \mathcal{C}_{j_1}(k) \mathcal{C}_{j_2}(k) e^{-i\eta(k_{j_1}+k_{j_2})\hat{z}_h} \sum_{s_h \in I_h} \rho_{s_h} E_x^{inc}\left(\boldsymbol{P}_{s_h}; k_{j_1}\right) E_x^{inc}\left(\boldsymbol{P}_{s_h}; k_{j_2}\right) \\
&= \sum_{h=1}^{H} e^{i\eta 2k\hat{z}_h} \sum_{j_1=1}^{L} \sum_{j_2=1}^{L} \frac{k^2}{k_{j_1} k_{j_2}} \mathcal{C}_{j_1}(k) \mathcal{C}_{j_2}(k) e^{-i\eta(k_{j_1}+k_{j_2})\hat{z}_h} \alpha_{cross}(k_{j_1}, k_{j_2}, h)
\end{aligned}$$

where $\alpha_{cross}(k_{j_1}, k_{j_2}, h) := \sum_{s_h \in I_h} \rho_{s_h} E_x^{inc}\left(\boldsymbol{P}_{s_h}; k_{j_1}\right) E_x^{inc}\left(\boldsymbol{P}_{s_h}; k_{j_2}\right)$ are called *cross-modal coefficients*.

3. SOLUTION TO THE MSR APPROXIMATION

In this section we explain how we solved Eq. (11) of the primary manuscript. To avoid confusion with the different wavenumbers we are going to use, we call ξ the *arbitrary* wavenumber and k_j the set of wavenumbers for which Eq. (6b) of the primary manuscript has been calculated. The variable β in Eq. (11) of the primary manuscript is bounded in $[-B, B]$ because otherwise the phase, βk, could be arbitrarily high, which would make the problem impossible to solve. We choose L wavenumbers $k_1, k_2, ..., k_L$ equally spaced that cover the spectrum. Then, for an arbitrary ξ in the spectrum, we select the subset of the T *closest* wavenumbers $k_{1_\xi}, ..., k_{T_\xi}$ to ξ (see Fig. (S1)), and we set $\mathcal{C}_j(\xi) = 0$ for all other k_j. To find a solution of Eq. (11) of the primary manuscript, we form an integral least square minimisation problem:

$$Loss(x_1, x_2, ..., x_T; \xi) := \int_{\mathbb{R}} \left[\int_{-B}^{B} \left| e^{-a^2\xi^2} e^{ib\xi} - \sum_{j=1}^{T} x_j e^{-a^2 k_{j_\xi}^2} e^{ibk_{j_\xi}} \right|^2 db \right] da, \tag{S2a}$$

$$\left[\mathcal{C}_{1_\xi}(\xi), ..., \mathcal{C}_{T_\xi}(\xi)\right] := \underset{[x_1,...,x_T] \in \mathbb{C}^T}{\operatorname{argmin}} \left\{ Loss(x_1, x_2, ..., x_T; \xi) \right\} \tag{S2b}$$

$$\mathcal{C}_j(\xi) := 0, \qquad \text{if } j \neq 1_\xi, ...T_\xi \tag{S2c}$$

The minimum in Eq. (S2b) is estimated by using the gradient descent method [1], for each wavenumber ξ. In order to avoid to recalculate Eqs. (S2) for each simulation, the functions $\mathcal{C}_j(\xi)$ are calculated and stored for several values of ξ and then loaded and interpolated over the spectrum of the simulation. Then, functions $\mathcal{C}_j(\xi)$ are employed in Eq. (13) of the primary document to rapidly estimate the DWI for each wavenumber.

4. MSR: PARAMETERS SETTING

The number of functions (L) employed by the method MSR, needed to reach a satisfactory approximation, increases with the choice of the upper bound B, which implies an increase in computational load. For this reason, an efficient way to solve the problem in Eq.(11) of the primary document is to find the smallest upper bound B for the phase variable β.

In our context, the term $e^{ik\beta}$ represents the phase term $e^{ik(b(\boldsymbol{s})-\eta\Delta)}$ in Eq.(12a) of the primary

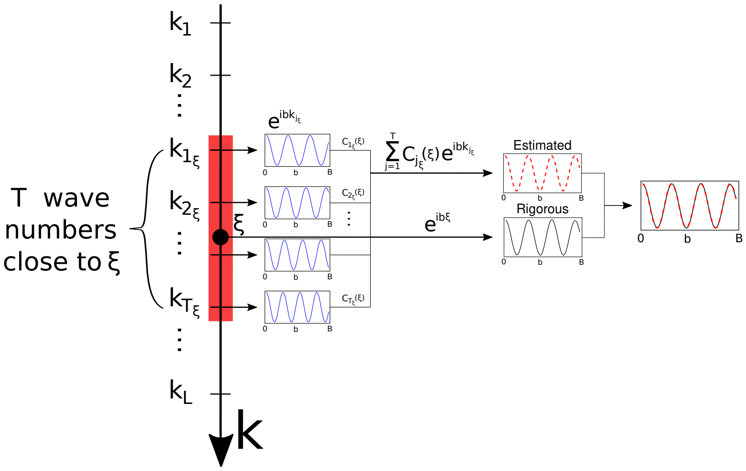

Fig. S1. Scheme of the MSR method (see Eq. S2), in the simplified case where only the phase terms are considered ($\alpha = 0$) in Eq. (11) of the primary document. ξ is an arbitrary wavenumber, $k_1, k_2, ...k_L$ are the set of the L equally-spaced wavenumbers in the spectrum, and $k_{1_\xi}, k_{2_\xi}, ..., k_{T_\xi}$ are the subset of T closest wavenumbers to ξ.

document, where $\Delta = \hat{z}_h - z$, $b(\boldsymbol{s}) = \eta \left(\boldsymbol{\rho} \cdot \boldsymbol{s} + z \left(\sqrt{1 - |\boldsymbol{s}|^2} - 1 \right) \right)$, and $|\boldsymbol{s}| \leq \frac{NA}{\eta}$.
We make use of the following inequality:

$$\left| \sqrt{1 - x^2} - 1 \right| \leq x^2, \qquad \forall x \in [0, 1]$$

An upper bound B for β is given by:

$$\begin{aligned} |\beta| &= \left| \eta \left(\boldsymbol{\rho} \cdot \boldsymbol{s} + z \left(\sqrt{1 - |\boldsymbol{s}|^2} - 1 \right) + \hat{z}_h - z \right) \right| \\ &\leq \eta \left(|\boldsymbol{\rho}||\boldsymbol{s}| + |z| \left| \sqrt{1 - |\boldsymbol{s}|^2} - 1 \right| + |\hat{z}_h - z| \right) \\ &\leq \eta \left(|\boldsymbol{\rho}||\boldsymbol{s}| + |z| \, |\boldsymbol{s}|^2 + |\hat{z}_h - z| \right) \\ &\leq \eta \left(\rho_{max} \frac{NA}{\eta} + z_{max} \left(\frac{NA}{\eta} \right)^2 + \frac{D}{2} \right) \\ &= \rho_{max} NA + z_{max} \frac{NA^2}{\eta} + \eta \frac{D}{2} \qquad =: B, \end{aligned}$$

where ρ_{max} and z_{max} are respectively the maximum distance of the scatterers from the optical axis (distant scatterers can be considered negligible for the DWI) and the axial coordinate of the deepest scatterer, respectively.

REFERENCES

1. B. Shi and S. S. Iyengar, *Mathematical Theories of Machine Learning - Theory and Applications* (Springer Cham, 2020).
